# Incidence of Dysplasia in Obese vs Nonobese Patients With Nondysplastic Barrett Esophagus

**DOI:** 10.31486/toj.19.0038

**Published:** 2019

**Authors:** Ann Monardo, Jennifer McCullough

**Affiliations:** ^1^ Department of Internal Medicine, Ascension Genesys Hospital, Grand Blanc, MI; ^2^ Department of Research, Ascension Genesys Hospital, Grand Blanc, MI

**Keywords:** *Adenocarcinoma of esophagus*, *Barrett esophagus*, *dysplasia*, *obesity*, *population surveillance*

## Abstract

**Background:** Obesity is a known independent risk factor for both Barrett esophagus and esophageal adenocarcinoma. However, data about the effect of obesity on the risk of progression from nondysplastic Barrett esophagus to dysplasia or esophageal adenocarcinoma are lacking. The aim of this study was to evaluate whether obese patients with nondysplastic Barrett esophagus had a higher incidence of dysplasia development during routine surveillance than nonobese patients.

**Methods:** In a retrospective review, 1,999 patients who had a first diagnosis of nondysplastic Barrett esophagus made by esophagogastroduodenoscopy (EGD) at a single community hospital were tracked to their surveillance EGD 3 to 5 years later to evaluate for dysplasia (low grade, high grade, or adenocarcinoma). We compared the incidence of dysplasia development in obese patients (body mass index [BMI] ≥30 kg/m^2^) with nonobese patients (BMI <30 kg/m^2^).

**Results:** The sample population included 1,019 obese patients (51.0%) and 980 nonobese patients (49.0%) with nondysplastic Barrett esophagus. Their mean age was 56.5 ± 11.6 years, 1,228 (61.4%) were male, and 1,853 (92.7%) were Caucasian. At surveillance endoscopy performed at a mean follow-up of 3.7 years after their first EGD, 51 obese patients (incidence of 15.3 cases per 1,000 person-years, 95% confidence interval [CI], 11.5-19.9) and 15 nonobese patients (incidence of 4.6 cases per 1,000 person-years, 95% CI, 2.7-7.4) had developed dysplasia (*P*=0.0001).

**Conclusion:** We found a significant increase in the incidence of dysplasia development in obese patients with nondysplastic Barrett esophagus at 3- to 5-year follow-up compared to nonobese patients. This finding suggests that more frequent surveillance in obese patients with nondysplastic Barrett esophagus may be warranted for early detection of dysplasia.

## INTRODUCTION

Barrett esophagus is a known complication of chronic gastroesophageal reflux disorder (GERD); approximately 10% to 15% of patients with chronic GERD are diagnosed with Barrett esophagus.^1^ Esophageal adenocarcinoma is the most feared consequence of Barrett esophagus, and those with Barrett esophagus have a 30 to 40 times greater risk of developing esophageal adenocarcinoma.^1^ The incidence of esophageal adenocarcinoma significantly increased in the western world between the years 1977 and 2017 and now surpasses esophageal squamous cell carcinoma as the most common form of esophageal cancer in the United States.^2^ The disease is usually diagnosed at an advanced stage, making the prognosis of esophageal adenocarcinoma very poor, with a 5-year survival rate <20%.^3^ Because Barrett esophagus is the only known precursor to esophageal adenocarcinoma, research efforts have focused on screening, surveillance, and treatment of Barrett esophagus.

The most common risk factors for Barrett esophagus and esophageal adenocarcinoma are GERD, increasing age, male sex, white race, smoking, and obesity. Several studies have examined body mass index (BMI) as well as measures of abdominal obesity; Kramer et al demonstrated that high waist-to-hip ratio but not BMI is associated with increased risk of Barrett esophagus.^4-6^ In contrast to these findings relative to Barrett esophagus, both BMI and abdominal obesity are independent risk factors for esophageal adenocarcinoma. The risk of developing esophageal adenocarcinoma in individuals with a BMI ≥30 kg/m^2^ is an estimated 2.4 to 2.8 times higher than the risk for those with normal BMI (18.5–24.9 kg/m^2^).^7,8^ The carcinogenic mechanism by which obesity contributes to development of esophageal adenocarcinoma is not fully understood, but one proposal is that adipokines (cell signaling proteins released by adipose tissue) may alter esophageal mucosal injury healing in Barrett esophagus, leading to progression of the disease.^2^ Investigation into the relationship between obesity-associated biomarkers—including levels of serum glucose, insulin, and adipokines (adiponectin and leptin)—and the risk of esophageal adenocarcinoma found a positive association between the obesity-associated biomarkers and increased risk of progression from Barrett esophagus to esophageal adenocarcinoma.^9^

The progression from Barrett esophagus to adenocarcinoma is thought to occur in a stepwise fashion from nondysplastic Barrett esophagus, to low-grade dysplasia, to high-grade dysplasia, and eventually to adenocarcinoma.^10^ Several studies have aimed to determine the incidence of dysplasia and esophageal adenocarcinoma in patients with nondysplastic Barrett esophagus, and the reported incidence of esophageal adenocarcinoma ranges widely from 0.2% to 3.5% per year in these studies.^11-17^ The Barrett esophagus study, a large multicenter cohort study of patients with nondysplastic Barrett esophagus, demonstrated an overall incidence of esophageal adenocarcinoma of approximately 0.2% to 0.4% per year, with an incidence of low-grade dysplasia of approximately 3.6% per year.^1^ To our knowledge, no large cohort studies similar to the Barrett esophagus study have evaluated the effect of obesity on the incidence of dysplasia and esophageal adenocarcinoma in patients with nondysplastic Barrett esophagus. A systematic review and metaanalysis by Kubo et al synthesized results from 7 observational studies (the majority were case-control studies) and demonstrated a positive association between increased BMI and risk for esophageal adenocarcinoma.^7^ Thrift et al performed a Mendelian randomization study, thereby eliminating confounding variables and bias, that indicated people with a high genetic propensity for obesity have a higher risk of esophageal metaplasia and neoplasia compared to the nonobese and provided strong evidence that obesity is independently associated with esophageal adenocarcinoma.^18^ Although these reports provide a prediction for obesity's effect on the development of esophageal adenocarcinoma, research investigating the incidence of dysplasia and esophageal adenocarcinoma in obese vs nonobese patients in a cohort of patients with nondysplastic Barrett esophagus is needed.

Current guidelines supported by the major gastroenterological societies, including the American College of Gastroenterology, the American Society for Gastrointestinal Endoscopy, and the American Gastroenterological Association, recommend surveillance upper endoscopy at 3- to 5-year intervals following the diagnosis of nondysplastic Barrett esophagus.^19^ Endoscopic radiofrequency ablation is an established treatment for eradication of Barrett esophagus and is considered the standard of care for patients with high-grade dysplasia or residual Barrett esophagus tissue after resection of esophageal adenocarcinoma.^10^ Research in 2014 evaluated the effect of endoscopic radiofrequency ablation on neoplastic progression in patients with Barrett esophagus and low-grade dysplasia and demonstrated a 25% reduced rate of progression to high-grade dysplasia and esophageal adenocarcinoma compared to patients who underwent surveillance only.^10^

As mentioned previously, the high mortality associated with esophageal adenocarcinoma is principally attributable to the late stage of the disease at diagnosis, emphasizing the importance of early detection of dysplasia to permit intervention that prevents progression. Although the correlation between obesity, Barrett esophagus, and esophageal adenocarcinoma has been established, data on the effect of obesity on the rate of progression from nondysplastic Barrett esophagus to dysplasia and esophageal adenocarcinoma are lacking.

We conducted a retrospective medical record review in a community hospital to analyze the incidence of dysplasia in a cohort of patients with nondysplastic Barrett esophagus. The aim of this study was to evaluate whether obese patients with nondysplastic Barrett esophagus had a higher incidence of dysplasia discovered during routine 3- to 5-year surveillance than nonobese patients. Our overall purpose was to explore the need for modified surveillance of obese patients with nondysplastic Barrett esophagus to ensure early detection of dysplasia and ultimately decrease their mortality.

## METHODS

This study was a single-center retrospective cohort study conducted at a 410-bed community teaching hospital. Institutional review board approval was obtained.

### Participants

Patients who had an initial diagnosis of nondysplastic Barrett esophagus made by esophagogastroduodenoscopy (EGD) between the years 2002 and 2018 were identified using the hospital's electronic medical record system. Inclusion criteria for the study were as follows: (1) patients 18 to 75 years who had been diagnosed with nondysplastic Barrett esophagus, that is, the presence of columnar-lined mucosa in the distal esophagus of ≥1 cm on endoscopy with the presence of intestinal metaplasia but no dysplasia confirmed on histology and (2) endoscopic follow-up 3 to 5 years from initial diagnosis in accordance with current surveillance guidelines.^19,20^

The following information was recorded for all patients included in the study: demographics (age, sex, BMI, and ethnicity), EGD data (date of initial diagnostic procedure and date of subsequent EGD), and diagnosis made at each EGD. When available in the medical record, smoking history and data on the patients’ use of aspirin, nonsteroidal antiinflammatory drugs (NSAIDs), proton pump inhibitors, and statins were recorded. Patients were divided into 2 groups based on their BMI: obese (BMI ≥30 kg/m^2^) and nonobese (BMI <30 kg/m^2^).

### Endoscopy, Histopathology, Surveillance

At initial EGD and surveillance EGD, each patient underwent biopsies of the Barrett segment. Because this study was conducted retrospectively, the biopsy protocol could not be standardized; however, the endoscopists documented biopsy protocols in their procedure reports consistent with the Seattle biopsy protocol. The Seattle biopsy protocol, a systematic 4-quadrant biopsy in which specimens are obtained at intervals of 2 cm, is the standard protocol for diagnosing Barrett esophagus.^21^ The endoscopic component of diagnosis is the presence of ≥1 cm columnar-lined mucosa in the distal esophagus. This protocol was used for both the index EGD and the surveillance EGD. Pathologists conducted histopathologic assessment of the biopsies, evaluating the specimens to confirm the presence of metaplasia (Barrett esophagus) and to evaluate for the presence and degree of dysplasia to make the diagnoses of nondysplastic Barrett esophagus, low-grade dysplasia, high-grade dysplasia, and esophageal adenocarcinoma.

### Statistical Analysis

The sample size was calculated based on an overall population rate of dysplasia of approximately 3% and a predicted moderately higher risk among the obese population vs the nonobese population.^1^ A 30% relative difference at power >80% required a sample size of approximately 1,000 per group or 2,000 patients total. Comparisons between the 2 groups were analyzed using chi-square analysis (confidence interval [CI]=95%).

## RESULTS

A total of 1,999 patients (1,019 obese patients [51.0%] and 980 nonobese patients [49.0%] met the inclusion criteria and were included in the study. Their mean age was 56.5 ± 11.6 years, and the majority (92.7%) were Caucasian. The cohort included 61.4% males, and the mean follow-up period was 3.7 years. Table 1 presents the demographic and baseline characteristics for the entire cohort and for the 2 comparison groups.

**Table 1.** Patient Demographics and Baseline Characteristics

		Nonobese Patients	Obese Patients
	All Patients	(BMI <30 kg/m^2^)	(BMI ≥30 kg/m^2^)
Variable	n=1,999	n=980	n=1,019
Age, years, mean ± SD	56.5 ± 11.6	56.4 ± 11.6	56.5 ± 11.5
Sex			
Male	1,228 (61.4)	616 (62.9)	612 (60.1)
Female	771 (38.6)	364 (37.1)	407 (39.9)
Race			
Caucasian	1,853 (92.7)	915 (93.4)	938 (92.1)
Non-Caucasian	146 (7.3)	65 (6.6)	81 (7.9)
BMI, kg/m^2^, mean ± SD	29.4 ± 5.5	25.2 ± 2.9	33.5 ± 4.1
Comorbidity/medication			
Smoking	425 (21.3)	198 (20.2)	227 (22.3)
Proton pump inhibitor	451 (22.6)	202 (20.6)	249 (24.4)
Aspirin	176 (8.8)	66 (6.7)	110 (10.8)
Statin	152 (7.6)	60 (6.1)	92 (9.0)
NSAID	92 (4.6)	34 (3.5)	58 (5.7)

Note: Data are presented as n (%) unless otherwise indicated.

BMI, body mass index; NSAID, nonsteroidal antiinflammatory drug.

### Incidence of Dysplasia and Esophageal Adenocarcinoma

At surveillance endoscopy 3 to 5 years after their first EGD demonstrating nondysplastic Barrett esophagus, 51 obese (5.0%) vs 15 nonobese (1.5%) patients had developed dysplasia, translating to a calculated incidence rate of dysplasia of 15.3 cases per 1,000 person-years (95% CI, 11.5-19.9) in the obese population vs 4.6 cases per 1,000 person-years (95% CI, 2.7-7.4) in the nonobese population. This difference was significant (*P*=0.0001). Of the 66 patients who developed dysplasia, 77.3% were obese.

Our population demonstrated an overall annual incidence of dysplasia (low-grade dysplasia, high-grade dysplasia, and esophageal adenocarcinoma) during the 5-year follow-up period of 0.98% per year (95% CI, 1.6-2.5) with an incidence of 3.06% per year (95% CI, 2.3-3.9) among the obese patients and 0.92% per year (95% CI, 0.5-1.5) among the nonobese patients (data not shown).

Among the 51 obese patients who developed dysplasia, 35 patients developed low-grade dysplasia, resulting in an incidence rate of 10.5 cases per 1,000 person-years (95% CI, 7.4-14.4). Fifteen obese patients developed high-grade dysplasia, resulting in an incidence rate of 4.5 cases per 1,000 person-years (95% CI, 2.6-7.3). One patient in the obese population developed esophageal adenocarcinoma, for a calculated incidence rate of 0.3 case per 1,000 person-years (95% CI, 0.02-1.5). The annual incidence among obese patients of developing low-grade dysplasia was 2.1% (95% CI, 1.5-2.9), the incidence for developing high-grade dysplasia was 0.9% (95% CI, 0.5-1.5), and the incidence of developing esophageal adenocarcinoma was 0.06% (95% CI, 0.03-2.9) (data not shown).

In comparison, among the 15 nonobese patients who developed dysplasia, 12 patients developed low-grade dysplasia, resulting in an incidence rate of 3.7 cases per 1,000 person-years (95% CI, 1.9-6.2); 3 patients developed high-grade dysplasia, resulting in an incidence rate of 0.9 case per 1,000 person-years (95% CI, 0.23-2.5); and no nonobese patients developed esophageal adenocarcinoma.

The numbers of cases and incidence rates are shown in Table 2.

**Table 2.** Body Mass Index (BMI)-Based Incidence of Dysplasia

			Nonobese Patients		Obese Patients	
	All Patients		(BMI <30 kg/m^2^)		(BMI ≥30 kg/m^2^)	
	n=1,999		n=980		n=1,019	
		Incidence rate		Incidence rate		Incidence rate
		per 1,000		per 1,000		per 1,000
	Number	person-years	Number	person-years	Number	person-years
Diagnosis	of cases	(95% CI)	of cases	(95% CI)	of cases	(95% CI)
Low-grade dysplasia	47	7.1 (5.3-9.4)	12	3.7 (1.9-6.2)	35	10.5 (7.4-14.4)
High-grade dysplasia	18	2.7 (1.6-4.2)	3	0.9 (0.23-2.5)	15	4.5 (2.6-7.3)
Esophageal adenocarcinoma	1	0.15 (0.008-0.75)	0		1	0.3 (0.02-1.5)
Total	66		15		51	

CI, confidence interval.

### Incidence of Dysplasia Development Based on Severity of Body Mass Index

Among the obese patients who developed dysplasia, 31 (60.8%) were class 1 obesity (BMI 30.0-34.9 kg/m^2^), 13 (25.5%) were class 2 obesity (BMI 35.0-39.9 kg/m^2^), and 7 (13.7%) were class 3 obesity (BMI ≥40.0 kg/m^2^).

Among the nonobese patients who developed dysplasia, 10 (66.7%) were overweight (BMI 25.0-29.9 kg/m^2^), 4 (26.7%) were normal weight (BMI 18.5-24.9 kg/m^2^), and 1 (6.7%) was underweight (BMI <18.5 kg/m^2^).

### Other Predictors of Progression to Dysplasia

Of the 66 patients who developed dysplasia at their first follow-up, 31 (47%) were current or past smokers, 33 (50%) patients were on a daily proton pump inhibitor, 11 (16.7%) patients were on a daily aspirin regimen, 10 (15.2%) patients were taking a daily NSAID, and 17 (25.8%) were on a daily statin.

In the subset of the 51 obese patients who developed dysplasia, 24 (47.1%) were current or past smokers, 24 (47.1%) were on a daily proton pump inhibitor, 8 (15.7%) were on a daily aspirin regimen, 7 (13.7%) were taking a daily NSAID, and 13 (25.5%) were on a daily statin.

## DISCUSSION

Large variation has been reported in the incidence rates of progression to dysplasia and esophageal adenocarcinoma among patients with nondysplastic Barrett esophagus, and no data are available on the effect of obesity on disease progression. Results of our single-center cohort of patients with nondysplastic Barrett esophagus demonstrated a significantly greater incidence of dysplasia development in obese patients vs nonobese patients at a mean follow-up period of 3.7 years (5% vs 1.5%, respectively). We found an overall incidence rate of dysplasia of 3.06% per year among the obese patients and 0.92% per year among the nonobese patients. Among the obese patients who developed dysplasia, the annual incidence rates were 0.06% for esophageal adenocarcinoma, 0.9% for high-grade dysplasia, and 2.1% for low-grade dysplasia. In comparison, the Barrett esophagus study reported annual incidence rates of 0.27% for esophageal adenocarcinoma, 0.48% for high-grade dysplasia, and 3.6% for low-grade dysplasia in a cohort of patients with nondysplastic Barrett esophagus.^1^

Prior to our study, no cohort studies in patients with nondysplastic Barrett esophagus provided a comparison of progression rates to dysplasia and esophageal adenocarcinoma in obese vs nonobese patients. Our study is unique in that it specifically evaluated a cohort of patients with nondysplastic Barrett esophagus, and we aimed to determine the incidence of dysplasia and not solely esophageal adenocarcinoma. Our results indicate a significantly greater incidence of dysplasia in obese patients vs nonobese patients at routine surveillance, which is the outcome we predicted.

Our study provides an estimate of the rate of progression to the endpoint of dysplasia (low-grade dysplasia, high-grade dysplasia, or esophageal adenocarcinoma) in obese patients with nondysplastic Barrett esophagus. Because endoscopic RFA provides a well-tolerated and effective means of preventing esophageal adenocarcinoma in patients with dysplasia, the results of this study are valuable. A 2010 study of patients with low-grade dysplasia indicated that progression to high-grade dysplasia or esophageal adenocarcinoma occurs at a rate of 13.4% per person-year.^22^ This remarkable risk of progression reinforces the importance of early detection of low-grade dysplasia.

One systematic review investigated the cost-effectiveness of surveillance in patients with nondysplastic Barrett esophagus and concluded that endoscopic surveillance is unlikely to be cost-effective.^23^ However, this review primarily included studies that did not consider the use of nonsurgical strategies such as radiofrequency ablation. Since the review was published, the use of radiofrequency ablation has grown rapidly and would likely significantly improve the quality-adjusted survival benefit conferred by endoscopic surveillance.

Several limitations of our study warrant consideration. Because of the retrospective evaluation, the biopsy protocol was not standardized. Pathology readings were not performed by the same pathologist, and we could not confirm that cases were reviewed by an expert gastrointestinal pathologist. Additionally, the participants’ BMI was recorded only once at their initial diagnosis and was not reassessed during surveillance, so we were unable to account for the possibility of a significant change in body mass. Because this study was observational, the results may be influenced by unmeasured confounders. Factors such as diet and exercise are likely related to BMI, but we did not adjust for them.

This study demonstrates significant data to support the positive association between obese BMI and the development of dysplasia and esophageal adenocarcinoma. Further studies, however, are needed to determine the mechanism by which obesity plays this role in cancer development. Additionally, although our study demonstrated increased rates of dysplasia development in obese patients, no studies have investigated whether weight loss in this same population could regress a patient's risk of esophageal adenocarcinoma.

## CONCLUSION

We found a significant increase in the incidence of dysplasia development in obese patients with nondysplastic Barrett esophagus at the 3- to 5-year follow-up EGD compared to nonobese patients. Our results suggest that shortening the surveillance intervals for obese patients diagnosed with nondysplastic Barrett esophagus should be considered. Although studies have demonstrated that EGD surveillance leads to earlier detection of dysplasia/esophageal adenocarcinoma and improved survival, no recommendations have been published to modify surveillance frequency in high-risk populations such as obese patients. The development of guidelines for surveillance endoscopy in nondysplastic Barrett esophagus that incorporates risk-stratification based on specific major risk factors such as obesity should be prioritized.

## Notes

This article meets the Accreditation Council for Graduate Medical Education and the American Board of Medical Specialties Maintenance of Certification competencies for Patient Care and Medical Knowledge.
